# Motor features in posterior cortical atrophy and their imaging correlates^☆^

**DOI:** 10.1016/j.neurobiolaging.2014.05.028

**Published:** 2014-12

**Authors:** Natalie S. Ryan, Timothy J. Shakespeare, Manja Lehmann, Shiva Keihaninejad, Jennifer M. Nicholas, Kelvin K. Leung, Nick C. Fox, Sebastian J. Crutch

**Affiliations:** a Dementia Research Centre, Department of Neurodegenerative Disease, University College London (UCL) Institute of Neurology, Queen Square, London, UK; b Department of Medical Statistics, Faculty of Epidemiology and Population Health, London School of Hygiene and Tropical Medicine, Keppel St, London, UK

**Keywords:** Posterior cortical atrophy, Corticobasal syndrome, Alzheimer's disease, Phenotype, Asymmetric atrophy

## Abstract

Posterior cortical atrophy (PCA) is a neurodegenerative syndrome characterized by impaired higher visual processing skills; however, motor features more commonly associated with corticobasal syndrome may also occur. We investigated the frequency and clinical characteristics of motor features in 44 PCA patients and, with 30 controls, conducted voxel-based morphometry, cortical thickness, and subcortical volumetric analyses of their magnetic resonance imaging. Prominent limb rigidity was used to define a PCA-motor subgroup. A total of 30% (13) had PCA-motor; all demonstrating asymmetrical left upper limb rigidity. Limb apraxia was more frequent and asymmetrical in PCA-motor, as was myoclonus. Tremor and alien limb phenomena only occurred in this subgroup. The subgroups did not differ in neuropsychological test performance or apolipoprotein E4 allele frequency. Greater asymmetry of atrophy occurred in PCA-motor, particularly involving right frontoparietal and peri-rolandic cortices, putamen, and thalamus. The 9 patients (including 4 PCA-motor) with pathology or cerebrospinal fluid all showed evidence of Alzheimer's disease. Our data suggest that PCA patients with motor features have greater atrophy of contralateral sensorimotor areas but are still likely to have underlying Alzheimer's disease.

## Highlights

• We investigated motor features and their neuroimaging correlates in 44 posterior cortical atrophy (PCA) patients.
• A total of 30% had asymmetrical left upper limb rigidity and were termed the “PCA-motor” group.
• Limb apraxia was more frequent and asymmetrical in PCA-motor, as was myoclonus.
• PCA-motor had greater asymmetry of atrophy, involving the right sensorimotor areas.
• The subgroup with pathology or cerebrospinal fluid all showed evidence of Alzheimer's disease.

## 1 Introduction

Posterior cortical atrophy (PCA) is a neurodegenerative syndrome characterized by progressive decline in visual processing, literacy, numeracy, and other functions that depend on parietal, occipital, and occipitotemporal brain regions (Benson et al., 1988, Kas et al., 2011). Most individuals with PCA have Alzheimer's disease (AD) as the underlying pathology, but PCA may also be caused by corticobasal degeneration (CBD), dementia with Lewy bodies (DLB), and Creutzfeldt-Jakob disease (CJD) (Renner et al., 2004, Tang-Wai et al., 2003a, Tang-Wai et al., 2003b). Although a relatively homogenous syndrome, there remains scope for improving the classification of PCA through consensus criteria and establishing clinicopathological correlations (Crutch et al., 2012). There is a need for a clearer description of the relationship and overlap between PCA and other related syndromes, particularly the corticobasal syndrome (CBS). Although commonly considered a selective visual syndrome, a number of patients with PCA develop sensorimotor signs, often asymmetric, which are more typically seen in CBS (Giorelli et al., 2014, McMonagle et al., 2006, Seguin et al., 2011, Tang-Wai et al., 2003a, Whitwell et al., 2010). In the original proposed criteria for PCA, a normal physical examination is considered supportive of a diagnosis but not mandatory (Mendez et al., 2002). Another proposed criteria excludes early Parkinsonism but recognizes that it may subsequently develop and does not state how early on its presence is acceptable (Tang-Wai et al., 2004). The prevalence of motor features in PCA is currently unclear, as are other potential differences between PCA patients with and without such signs.

The core clinical features of CBS are progressive asymmetrical limb rigidity and apraxia (Boeve et al., 2003). There may be additional cortical signs such as myoclonus, alien limb phenomenon and cortical sensory loss and extrapyramidal signs including tremor, bradykinesia, and dystonia. All these signs contribute to the patient experiencing clumsiness and loss of function of the affected limb. Historically, these clinical features were considered to be diagnostic of CBD (a 4-repeat tau neurodegenerative disease) (Dickson et al., 2002, Rebeiz et al., 1968), however, numerous clinicopathological studies have demonstrated that CBD can cause a variety of different phenotypes including progressive supranuclear palsy syndrome, frontotemporal dementia, progressive nonfluent/agrammatic aphasia, an executive-motor syndrome, and PCA (Armstrong et al., 2013, Josephs et al., 2006, Kertesz et al., 2000, Lee et al., 2011). Furthermore, it has become apparent that CBD pathology is found at autopsy in less than half of the patients who are clinically diagnosed during life (Boeve et al., 1999, Josephs et al., 2006, Lee et al., 2011). Increasingly, AD is recognized as an important cause of CBS but other alternative pathologies include progressive supranuclear palsy, Pick's disease, frontotemporal lobar degeneration with TAR DNA binding protein inclusions, DLB, and CJD (Boeve et al., 1999, Hu et al., 2009, Josephs et al., 2006, Ling et al., 2010, Shelley et al., 2009). The term “CBS” was therefore coined to refer purely to the constellation of clinical features, in recognition that they may be caused by diverse underlying pathologies (Boeve et al., 2003, Kertesz et al., 2000).

CBS terminology has been in circulation for over a decade and during this time, there has been growing appreciation of the cognitive dysfunction that may feature in the disorder (Rabinovici and Miller, 2012). Cognitive function was initially reported to be relatively well preserved until late in the disease and initial diagnostic criteria made early dementia an exclusion criterion (Lang et al., 1994, Rebeiz et al., 1968). However, increasing numbers of studies reported cognitive symptoms early in the course of CBS or even predating the emergence of motor features (Graham et al., 2003, Grimes et al., 1999, Kertesz et al., 2000, Murray et al., 2007, Schneider et al., 1997). Although no consensus yet exists for the diagnosis of CBS, proposed criteria have subsequently included cognitive dysfunction as a supportive feature (Boeve et al., 2003) or have given equal weight to the cognitive and motor aspects of the disorder (Mathew et al., 2012). In addition to limb apraxia, the characteristic cognitive manifestations of CBS are frontal dysfunction and language impairment (typically nonfluent aphasia), but also dysgraphia, dyscalculia, and visuospatial deficits (Graham et al., 2003, Mathew et al., 2012). The relationship between CBS, frontotemporal lobar degeneration, and primary progressive aphasia has been the subject of a number of studies (Josephs et al., 2006, Kertesz et al., 2000), however, the overlap between CBS and PCA has received comparatively little attention. The aims of this study were to ascertain how frequently the core features of CBS were observed in a relatively large cohort of PCA patients and compare the clinical and neuroimaging profiles of PCA patients with and without these signs. In line with previous evidence of an association between CBS and perirolandic atrophy irrespective of underlying pathology (Lee et al., 2011), we hypothesized that PCA patients with motor features would show greater atrophy of contralateral sensorimotor cortices.

## 2 Methods

### 2.1 Subject characteristics

The study was conducted at the Dementia Research Centre (DRC), University College London Institute of Neurology, at the National Hospital for Neurology and Neurosurgery. Most of the subjects in this study had attended our Specialist Cognitive Disorders Clinic for their clinical assessment. The DRC database was interrogated to identify individuals with a clinical diagnosis of PCA and at least 1 magnetic resonance imaging (MRI) brain scan and a neurologist then reviewed their clinical notes. Subjects were included if they met the original proposed clinical criteria for PCA (Mendez et al., 2002), of presentation with progressive visual complaints, intact primary visual functions, and a predominant complex visual disorder on examination, with proportionally less impaired memory and insight. They were only included in this study if they had a clearly documented neurological examination within a year of their MRI scan. A total of 44 individuals with PCA were identified, along with 30 healthy controls. Informed consent was obtained from all subjects according to the Declaration of Helsinki and the study had local ethics committee approval. Some of the patients had been included in preceding studies from our group (Crutch et al., 2011, Crutch et al., 2013a; Lehmann et al., 2011a, Lehmann et al., 2011b, Lehmann et al., 2012, Kennedy et al., 2012, Ridgway et al., 2012, Shakespeare et al., 2013, Witoonpanich et al., 2013, Yong et al., 2013).

The same neurologist reviewed all the clinical assessment notes to ascertain the age at symptom onset, mini-mental state examination (MMSE) (Folstein et al., 1975) score and the presence of limb rigidity, apraxia, myoclonus, tremor, dystonia, and alien limb phenomenon at the time of scanning. Not all patients were consistently examined for cortical sensory signs or bradykinesia so these features were not considered for the purpose of this study. If the motor signs were observed on clinical examination to affect one side only or one side more than the other, this asymmetry was documented. Limb rigidity is a core feature shared by all 3 of the existing diagnostic criteria for CBS (Boeve et al., 2003, Lang et al., 1994, Mathew et al., 2012) and was used to define the group membership for this study. Patients were classified as PCA-motor if prominent limb rigidity was present or PCA-pure if absent. They were not included in the PCA-motor group if a very subtle increase in limb tone was only evident on testing with synkinesis.

Six patients had undergone lumbar puncture (LP). Cerebrospinal fluid (CSF) was analyzed for 14-3-3 protein positivity and total tau and amyloid beta (Aβ_1-42_) concentrations (Innotest platforms, Innogenetics, Ghent, Belgium). According to our laboratory's local reference ranges, a CSF profile is considered to show 85% sensitivity for a clinical diagnosis of AD when tau >307pg/mL and Aβ_1-42_ <325pg/mL (unpublished data). Two patients had come to postmortem and a third patient had undergone brain biopsy. DNA was available for 40 of the PCA patients, which was analyzed to establish apolipoprotein E (*APOE*) genotype.

### 2.2 Neuropsychological assessment

Detailed neuropsychological assessment was available for 21/31 (67%) PCA-pure and 9/13 (69%) PCA-motor patients. This included tests of visuospatial processing (number location and dot counting from the Visual Object and Space Perception battery; Warrington and James, 1991), visuoperceptual processing (fragmented letters and object decision from the Visual Object and Space Perception battery), calculation (Graded Difficulty Arithmetic test; Jackson and Warrington, 1986), spelling (Baxter Graded Difficulty Spelling test; Baxter and Warrington, 1994), verbal and visual memory (Short Recognition Memory Test for words and faces (Warrington, 1996)), naming from verbal description and praxis (gesture production in the dominant upper limb, a subset of 5 of the traditional gesture tasks from Crutch et al., 2007).

The limb apraxia subtest of the Apraxia Battery for Adults (ABA-2,3A) (Dabul, 2000) was recently introduced into our clinical assessment protocol to provide a standardized assessment of limb praxis. Therefore, this subtest was available for only the 8 most recently assessed patients (4 PCA-motor, 4 PCA-pure). In this assessment, 10 different actions are verbally described and the subject is scored out of 5 to record how accurately they perform each gesture. We conducted the assessment separately for the right and left upper limb to give a total score of 50 for each limb, from which the difference between scores for left and right could be calculated.

### 2.3 MRI acquisition

T1-weighted volumetric MR brain scans were acquired on a 3.0 T Siemens TIM Trio scanner (N = 43, Siemens, Erlangen, Germany) using a magnetization prepared rapid gradient echo sequence with a 28.2 cm field of view to provide 208 contiguous 1.1 mm thick slices, and on 1.5 T GE Signa units (N = 31, General Electric, Milwaukee, WI, USA) using a spoiled gradient recalled sequence with a 24 cm field of view to provide 124 contiguous 1.5-mm thick slices. The proportion of patients scanned on each scanner was balanced across groups (Table 1). Full details of the scanner acquisition protocols can be found in the Supplementary Information.

**Table 1:** Subject characteristics and clinical data

	Controls (N = 30)	PCA-motor (N = 13)	PCA-pure (N = 31)	*p*-value
Gender (male, female)	13, 17	7, 6	12, 19	0.71^a^
Age in years, mean (SD)	63.9 (6.2)	63.8 (8.0)	63.4 (6.2)	0.94^b^
MMSE, mean (SD)	29.3 (0.8)	17.4 (6.3)	18.9 (6.4)	0.49^c^
Disease duration in years, mean (SD)	NA	4.9 (2.1)	5.3 (2.8)	0.66^c^
Scanner (3.0 T, 1.5 T)	18, 12	7, 6	18, 13	0.95^a^
APOE4, number (%) with *E4* allele/number with DNA sample	NA	5/11 (45)	12/29 (41)	1.00^a^
Neurological signs				
Limb rigidity, n (%)^d^^e^	NA	13 (100)	0	
Alien limb phenomena, n (%)^d^	NA	2 (15)	0	
Tremor, n (%)^d^	NA	3 (23)	0	
Myoclonus, n (%)^d^	NA	10 (77)	14 (45)	0.10^a^
Asymmetrical, n (%)		8 (80)	4 (29)	0.04^a^
Apraxia, n (%)^d^	N/A	13 (100)	12 (39)	<0.001^a^
Asymmetrical, n (%)		12 (92)	2 (17)	<0.001^a^
Limb apraxia subtest (3A) of apraxia battery for adults (ABA-2A)		PCA-motor (N = 4)	PCA-pure (N = 4)	
Right upper limb score^f^, mean (SD)	NA	34.4 (12.3)	42.75 (10)	0.32^c^
Left upper limb score^f^, mean (SD)	NA	21.3 (13.3)	42.75 (7.3)	0.05^c^
Difference between left and right scores, mean (SD)	NA	13 (5.9)	2 (2.3)	0.01^c^
*p*-Value for paired samples *t*-test comparing left and right upper limb scores		0.02	1.00	

Key: ANOVA, analysis of variance; MMSE, mini-mental state examination; NA, not applicable; PCA, posterior cortical atrophy; SD, standard deviation.

a Fisher exact test.

b One-way ANOVA.

c Two sample unpaired *t*-test comparing PCA-motor against PCA-pure.

d Numbers indicate the number (percentage) of patients in each group documented as manifesting the sign on clinical examination. Where indicated, the number (percentage) of these subjects in whom the sign was asymmetrical that is, observed only or more prominently on 1 side than the other, is recorded on the line below. The remaining signs were asymmetrical in all cases.

e Limb rigidity was the feature used to define membership of the PCA-motor group and affected the left upper limb in all cases.

f Maximum score 50, with lower scores indicating more severe apraxia.

### 2.4 Voxel-based morphometry processing

Voxel-based morphometry (VBM) was carried out using SPM8 (Statistical Parametric Mapping, version 8; Wellcome Trust Centre for Neuroimaging, London, UK). Scans were segmented into gray and white matter using SPM8's new segment toolbox with default settings (Ashburner and Friston, 2005, Weiskopf et al., 2011). Segmentations were produced with rigid alignment to standard space (Montreal Neurological Institute [MNI] space) and resampled to 1.5 mm isotropic voxels for use with DARTEL (Ashburner, 2007). DARTEL then iteratively registered the gray and white matter segments to an evolving estimate of their group-wise average (Ashburner and Friston, 2009). The native space tissue segments were then normalized to MNI space using the DARTEL transformations, modulated to account for local volume changes. A 6 mm full width at half maximum Gaussian smoothing kernel was applied. Total intracranial volume (TIV) for each participant was estimated using Jacobian integration of deformation fields. An explicit mask was applied to include only voxels for which the intensity was at least 0.1 in at least 80% of the images (Ridgway et al., 2009).

### 2.5 Cortical thickness processing

Cortical thickness measurements were made using FreeSurfer version 5.1.0 (http://surfer.nmr.mgh.harvard.edu/). The detailed procedure has been described and validated in previous publications (Dale et al., 1999, Fischl and Dale, 2000). Two modifications to the standard FreeSurfer processing stream were made: a locally generated brain mask was used to improve skull stripping, and FreeSurfer ventricular segmentations were added to the white matter mask to improve cortical segmentation.

### 2.6 Whole-brain analysis of VBM and cortical thickness data

A general linear model was used to assess group differences in gray and white matter volume in the VBM analysis (implemented in SPM) and cortical thickness in the FreeSurfer analysis (implemented using SurfStat, http://www.math.mcgill.ca/keith/surfstat/ (Chung et al., 2010) and a locally-developed Matlab toolkit). Volume and cortical thickness were modeled as a function of group (controls, PCA-pure, and PCA-motor), adjusting for age, gender, TIV (all mean centered) and scanner. Statistical significance of differences between groups was tested using family-wise error (FWE) correction at *p* < 0.05. Maps showing statistically significant differences between the controls and patient groups as well as maps showing percent differences between the 2 patient groups were generated.

### 2.7 Cortical region of interest analysis

Cortical thickness values were extracted for 34 brain areas in the left and right hemisphere using FreeSurfer's Desikan parcellation (Desikan et al., 2006). These areas were grouped into 5 larger regions–central, frontal, parietal, temporal, and occipital (see Appendix). To investigate differences in laterality of cortical thickness between patient groups in all 5 regions, 6 linear regressions were performed (using Stata 12–StataCorp, 2011), 1 for each region of interest (ROI) and 1 for all ROIs combined. Cortical thickness was the dependent variable and group, hemisphere and their interaction were the independent variables of interest. Robust standard errors were used to account for repeated measures by patient. Age, gender, scanner, and TIV were included as additional covariates for adjustment. Wald tests were carried out to elucidate the main effects of group and laterality and their interaction. To compare the size of the effect of differences between PCA-motor and PCA-pure groups, Cohen's d was calculated for this comparison in each of the cortical ROIs in the right and left hemisphere.

### 2.8 Subcortical ROI analysis

The Multi-Atlas Propagation and Segmentation (MAPS) technique was used to investigate volumes of the subcortical structures of interest in this study; namely the thalamus, caudate, and putamen. This segmentation method was previously developed for hippocampal segmentation (Leung et al., 2010) and has been used in brain extraction (Leung et al., 2011). In MAPS, the target T1-weighted image is compared with all the atlases in a template library, comprising 30 MRI scans of healthy individuals which have been manually segmented into 83 anatomic structures (Hammers et al., 2003). Multiple best-matched atlases were used to segment the target image, and an optimal segmentation was created by fusing the multiple segmentations. Leave-one-out cross-validation comparing the automated and manual segmentations of the template library was used to determine the optimal number of best-matched atlas (7 for putamen and thalamus and 9 for caudate) and label-fusion algorithm (simultaneous truth and performance level estimation) (Warfield et al., 2004). We used the optimized parameters to generate individual ROIs from MAPS for each subject. Linear regression analysis was used to test the effect of group, laterality, and their interaction in the same way as for the cortical thickness ROIs. Similarly, effect sizes were calculated using Cohen's d for the comparison between PCA-motor and PCA-pure.

## 3 Results

### 3.1 Clinical

The control and PCA groups were matched for age and gender (see Table 1 for demographics and clinical data). The patient subgroups were matched for age at scan, disease duration (time between symptom onset and scan), and MMSE score. There was no significant difference in *APOE4* allele frequency between the 2 PCA subgroups and 2 patients in each group were *E4* homozygous.

Of the 44 PCA patients, 13 (30%) met inclusion criteria for PCA-motor and 31 (70%) for PCA-pure. In all patients with limb rigidity (PCA-motor) the rigidity was asymmetrical, and in all 13 the left arm was predominantly affected. All 13 PCA-motor patients demonstrated apraxia on clinical examination, which was considered by the examining neurologist to be asymmetrical (all demonstrating worse gesture production using the left than right hand), in 92% of them. In the PCA-pure group, apraxia was observed in 39% and was described as asymmetrical in 17% of those with apraxia. Therefore, although limb apraxia occurred in both groups, it was seen significantly more frequently (*p* < 0.001) and was more frequently asymmetrical (*p* < 0.001) in the PCA-motor than PCA-pure group. On the ABA-2,3A test, the mean score in the 4 PCA-pure patients tested was 43/50 for both left and right upper limb, which is considered indicative of mild apraxia (Dabul, 2000), with a mean difference in scores between left and right of 2. In the 4 PCA-motor patients tested, the mean score was 34/50 for the right (moderate apraxia) and 21/50 (severe apraxia) for the left upper limb, with a mean difference in scores between left and right of 13. Although the subgroup of patients assessed with this battery was small, the PCA-motor group demonstrated significant asymmetry in the severity of their apraxia between left and right upper limb (*p* = 0.02), which was significantly worse than the degree of asymmetry observed in the PCA-pure group (*p* = 0.01).

Myoclonus was observed in both the PCA-motor (77%) and PCA-pure (45%) group but was more frequently asymmetrical in the PCA-motor group (*p* = 0.04). A total of 80% of those with myoclonus in the PCA-motor group had asymmetric myclonus, which was only or more prominently observed on the left. In the PCA-pure group, asymmetry was observed in 29% of those with myoclonus but half of this subgroup demonstrated worse myoclonus on the left and half demonstrated worse on the right. In the PCA-motor group, 3 subjects (23%) had a resting tremor of the left hand and 2 subjects (15%) had alien limb phenomena affecting the left upper limb. None of the subjects in the PCA-pure group had a rest tremor or signs of alien limb. No subjects were documented to have dystonia. Two of the PCA-motor subjects had symptoms of rapid eye movement sleep behavior disorder; both manifested myoclonus and one had signs of alien limb. No subject reported visual hallucinations. However, 1 PCA-motor subject had experienced extracampine hallucinations earlier on in his illness. He described feeling a presence on his left, with involuntary drifting of his left arm, which began 2 years after his cognitive symptoms and resolved 3 years later. No symptoms or signs of alien limb were evident at his assessment, 6 years into his illness, although he did have left upper limb rigidity, apraxia, myoclonus, and a resting tremor. The time of onset of motor features was not clearly documented or not known for most of the patients. However, 3/13 PCA-motor subjects had reported difficulty using the left hand from the onset of their illness.

One PCA-motor and 1 PCA-pure patient underwent postmortem pathological examination. Both had AD with Braak & Braak stage VI neurofibrillary pathology (the most severe grading and considered diagnostic) (Braak and Braak, 1995), which involved visual cortex. The PCA-motor case (the individual with extracampine hallucinations described previously) additionally had Lewy body pathology (LBP) in limbic and brainstem structures, and mild amyloid angiopathy. The PCA-pure case showed severe amyloid angiopathy but no LBP. Another PCA-motor patient underwent a right frontal lobe biopsy, which demonstrated AD pathology with amyloid angiopathy but no LBP.

CSF total tau and Aβ_1-42_ concentrations were analyzed for 6 other patients (2 PCA-motor, 4 PCA-Pure, see Table 2). All subjects with complete CSF analysis had increased concentrations of total tau supporting an underlying diagnosis of neurodegeneration. The majority (5/6) also had low concentrations of Aβ_1-42_ and a raised tau to Aβ_1-42_ ratio >1, supportive of a diagnosis of AD (Bian et al., 2008, Blennow and Hampel, 2003). The only patient with a particularly high Aβ_1-42_ concentration for AD (patient 1), nonetheless had a tau/Aβ_1-42_ ratio >1 and was in the PCA-pure group. This individual had a typical clinical presentation for PCA but was only very mildly affected (MMSE 29/30) at the time of his LP. None of the patients had a positive CSF 14-3-3 protein level.

**Table 2:** CSF results for the patients who underwent LP

	Patient 1	Patient 2	Patient 3	Patient 4	Patient 5	Patient 6
Group	PCA-pure	PCA-pure	PCA-pure	PCA-pure	PCA-motor	PCA-motor
Total tau (pg/mL)	931	Insufficient	488	660	630	566
Aβ_1-42_ (pg/mL)	625	105	135	343	243	205
Tau:Aβ ratio	1.49	NA	3.61	1.92	2.59	2.75

Key: CSF, cerebrospinal fluid; LP, lumbar puncture; NA, not applicable; PCA, posterior cortical atrophy.

### 3.2 Neuropsychology

There were no significant differences between the PCA-motor and PCA-pure groups on any neuropsychological test, including assessments of visual function and naming (see Table 3). There was, however, a trend toward worse performance in gesture production in the PCA-motor (mean 8.6, SD 4.16) than PCA-pure group (mean 11.6, SD 4.13, *p* = 0.08 between groups).

**Table 3:** Neuropsychology scores

	PCA-pure mean (SD)	PCA-motor mean (SD)	Unpaired *t* test (*p*-value)	Normative mean (SD)	PCA-pure number (%) below the 5th percentile	PCA-motor number (%) below the 5th percentile
Interval between MRI and psychology (months)	3.21 (4.25)	3.05 (6.04)	0.93	—	—	—
General function						
sRMT words (/25)^a^	19.2 (2.83)	20.3 (2.60)	0.30	23.5 (2.10)	17 (81.0)	5 (55.6)
sRMT faces (/25)^a^	18.6 (5.63)	15.5 (4.71)	0.30	22.8 (1.90)	4 (19.1)	4 (44.4)
Concrete synonyms (/25)^b^	19.4 (5.67)	20.8 (4.63)	0.54	20.8 (3.00)	3 (14.2)	1 (11.1)
Naming from description (/20)^c^	13.3 (6.77)	12.4 (4.69)	0.72	18.9 (1.50)	11 (52.4)	7 (77.8)
Gesture production	11.6 (4.13)	8.56 (4.16)	0.08	—	—	—
Non-visual parietal						
Calculation (adap. GDA/26)^d^	9.24 (5.20)	9.33 (5.24)	0.96	20.7 (3.10)	19 (90.5)	8 (88.9)
Spelling (Baxter/20)^e^	8.05 (6.71)	7.44 (7.23)	0.83	19.5 (6.49)	10 (47.6)	5 (55.6)
Visual function						
Figure-ground (/20)^f^	15.3 (3.73)	15.0 (4.00)	0.83	19.9 (0.30)	17 (81.0)	8 (88.9)
Fragmented letters (/20)^f^	3.72 (4.59)	1.11 (1.62)	0.11	18.8 (1.40)	17 (81.0)	9 (100)
Object decision (/20)^f^	10.6 (5.31)	9.56 (4.28)	0.61	17.7 (1.90)	14 (66.7)	9 (100)
Usual views (/20)^g^	13.7 (7.09)	7.75 (5.74)	0.16	19.7 (0.50)	6 (28.6)	4 (44.4)
Unusual views (/20)^g^	4.90 (4.82)	2.25 (1.89)	0.32	17.1 (3.00)	9 (42.9)	4 (44.4)
Number location (/20)^f^	3.06 (3.37)	1.44 (2.01)	0.20	9.40 (1.10)	15 (71.4)	9 (100)
Dot counting (/20)^f^	3.95 (3.73)	3.00 (3.57)	0.53	9.90 (0.20)	17 (81.0)	8 (88.9)

Mean and standard deviation of raw scores for the PCA patient group and relevant normative data.

Key: MRI, magnetic resonance imaging; PCA, posterior cortical atrophy; sRMT, Short Recognition Memory Test.

Normative data samples are as follows:

a Warrington (1996).

b Warrington et al. (1998).

c Randlesome (unpublished data N = 100).

d Crutch (unpublished data).

e Baxter and Warrington (1994).

f Warrington and James (1991).

g Warrington and James (1988).

### 3.3 Voxel-based morphometry analysis

#### 3.3.1 Gray matter

**Fig. 1:**
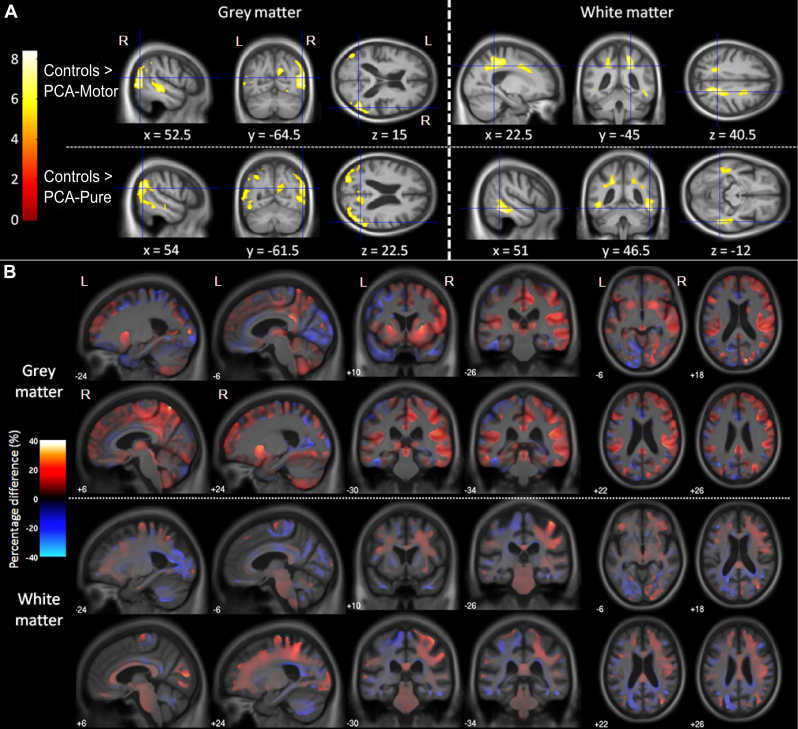
Results from voxel-based morphometry analysis. (A) Differences in gray and white matter volume between controls and PCA-motor, and between controls and PCA-pure. T-scores are shown for statistically significant lower gray matter volume in the patient groups compared with controls (FWE corrected at *p* < 0.05). Images shown in neurological convention (right on right). Cross hairs and coordinates (in MNI space) indicate t-score global maxima (this is in the right hemisphere for both comparisons). (B) Percent difference maps for differences in gray and white matter between PCA-motor and PCA-pure. Warmer colors show regions of lower volume in PCA-motor compared with PCA-pure, whereas cooler colors show regions for the opposite contrast. Images shown in neurological convention (right on right), coordinates are in MNI space. Abbreviations: FWE, family-wise error; PCA, posterior cortical atrophy.

The PCA-motor group showed lower gray matter volume in the occipital and parietal lobe regions compared with controls (peak in right lateral occipital cortex, MNI [52, −64.5, 15]), with some additional lateral temporal lobe involvement. On visual inspection, differences appeared greater in the right hemisphere than the left (Fig. 1A). A similar pattern of reduced gray matter volumes in occipital and parietal lobe regions was found in the PCA-pure group compared with controls (peak in right lateral occipital cortex, MNI [52, −61.5, 22.5]), however, asymmetry between hemispheres was less pronounced. The direct comparison of PCA-motor versus PCA-pure did not produce statistically significant differences after correcting for multiple comparisons (FWE *p* < 0.05). However, percent difference maps revealed regions of lower gray matter volume on the right in the PCA-motor group, particularly in the fronto-parietal operculum, supramarginal gyrus, and middle part of the cingulate gyrus, with lower left occipital volume suggested in the PCA-pure group (Fig. 1B).

#### 3.3.2 White matter

Effects in the white matter reflected those seen in the gray matter. The PCA-motor group showed lower white matter volume in bilateral parietal cortex compared with controls and also in the right frontal cortex. Again, visual inspection suggested a greater spatial extent of white matter involvement in the right hemisphere. The PCA-pure group showed reduced white matter volume in the parietal and temporal regions compared with controls but with less pronounced asymmetry. The direct PCA-motor versus PCA-pure comparison did not produce statistically significant differences after correcting for multiple comparisons (FWE *p* < 0.05). However, percent difference maps revealed regions of lower white matter volume in the right hemisphere in the PCA-motor group, particularly extending anteroposteriorly between the frontal and parietal cortices, with lower left occipital volume suggested in the PCA-pure group.

### 3.4 Cortical thickness analysis

#### 3.4.1 Whole-brain cortical thickness results

**Fig. 2:**
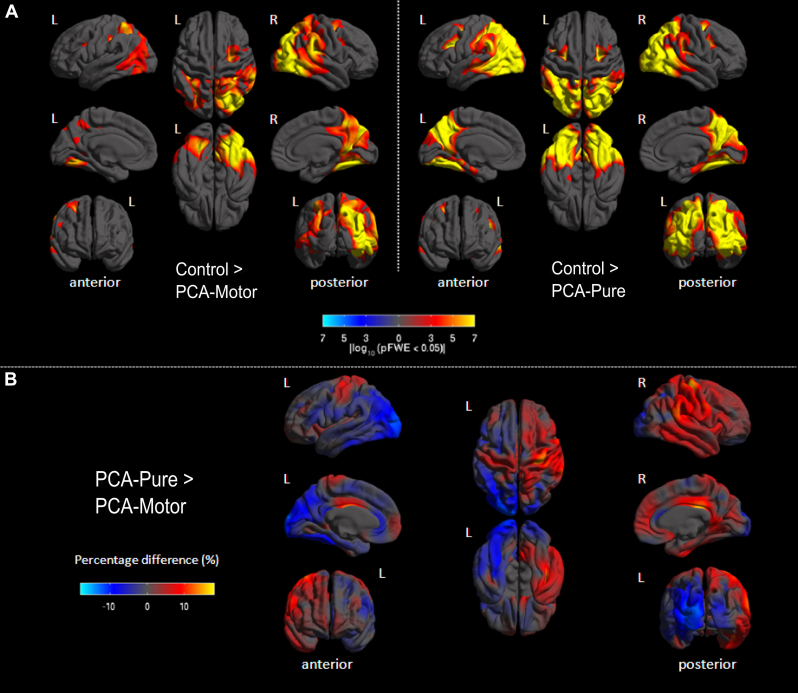
Results from cortical thickness analysis. (A) Differences in cortical thickness between controls and PCA-motor, and between controls and PCA-pure. The color scale represents FWE corrected *p*-values (*p* < 0.05), with warmer colors representing regions with lower cortical thickness in the patient groups compared with controls, and cooler colors showing the opposite contrast (which yielded no statistically significant results). (B) Difference in cortical thickness between PCA-pure and PCA-motor. The color scale represents percent differences in cortical thickness with warmer colors showing regions where PCA-motor has lower cortical thickness compared with PCA-pure, whereas cooler colors represent areas for the opposite contrast. Abbreviations: FWE, family-wise error; PCA, posterior cortical atrophy.

Comparing the 2 patient groups with controls revealed reduced cortical thickness predominantly in the occipital and parietal lobes, including the posterior parietal lobe, precuneus, posterior cingulate gyrus, as well as fusiform gyrus (Fig. 2A). On visual inspection, lower cortical thickness was found bilaterally in the PCA-pure group, whereas the PCA-motor group showed greater involvement in right hemisphere regions. The direct comparison of PCA-motor and PCA-pure did not reveal any statistically significant results after FWE correction. However, percent difference maps revealed trends toward lower cortical thickness in the left occipitoparietal regions of the PCA-pure group, whereas the PCA-motor group showed lower cortical thickness in the right hemisphere, including the motor cortex (Fig. 2B).

#### 3.4.2 Regional and lateralization cortical thickness results

**Fig. 3:**
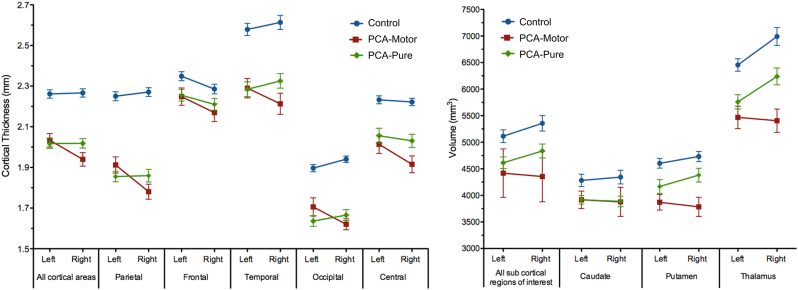
Mean cortical thickness and subcortical volume for each patient group in each region of interest. Error bars indicate standard error.

Mean cortical thickness of each region in the left and right hemisphere for each group is presented in Fig. 3. Combining all regions and both hemispheres, pairwise comparisons revealed lower global cortical thickness in the PCA-motor (mean 1.99 mm, SD 0.27) and PCA-pure group (mean 2.02 mm, SD 0.30) compared with controls (mean 2.26 mm, SD 0.25; *p* < 0.001 for both comparisons), but no evidence for an overall difference in cortical thickness between PCA-motor and PCA-pure (*p* = 0.73). This effect was comparable when looking at each cortical ROI separately and is evident in Fig. 3 (see Supplementary Table 1 for *p*-values and mean differences for pairwise comparisons).

There was, however, a significant interaction between hemisphere and group in every region (*p* < 0.002) except frontal cortex (*p* = 0.514). Thus, in all regions other than frontal cortex, the difference in cortical thickness between left and right hemispheres depended upon the patient group. Pairwise comparisons revealed that this was driven by more loss in right hemisphere regions in the PCA-motor than PCA-pure and control groups. In some regions (parietal, occipital, and temporal) this constituted a reversal of the asymmetry seen in controls, whereas in others (all cortical ROIs combined and central) there was asymmetry in PCA-motor, although there was no left-right difference in controls. There was no evidence for a difference in the laterality of cortical thickness between controls and the PCA-pure group in any cortical ROI.

### 3.5 Subcortical volumetric analysis

Pairwise comparisons for both individual and combined subcortical ROIs (caudate, putamen, and thalamus) are shown in Fig. 3 and Supplementary Table 1. Controls (combined mean = 5235 mm^3^, SD = 1262) showed higher volumes on all metrics than either PCA-pure patients (combined mean = 4726 mm^3^, SD = 1032; *p* = 0.001) or PCA-motor patients (combined mean = 4387 mm^3^, SD = 1032; *p* = 0.001). The PCA-motor group had significantly lower volumes than the PCA-pure group for the combined subcortical ROIs (*p* = 0.009), thalamus (*p* = 0.005), and putamen (*p* = 0.004) but not caudate (*p* > 0.9).

There was a significant interaction between hemisphere and group in all regions combined (*p* < 0.001), thalamus (*p* < 0.001), and putamen (*p* < 0.001) but not caudate (*p* = 0.075). As with the cortical regions of interest, pairwise comparisons revealed that this interaction was driven by lower volumes in right hemisphere regions in the PCA-motor group than PCA-pure and control groups. In all subcortical regions combined and the thalamus separately, controls and PCA-pure showed asymmetry with greater volume in the right hemisphere, although this asymmetry was absent in the PCA-motor group. The magnitude of thalamic asymmetry did not differ between controls and PCA-pure but both groups were significantly more asymmetrical than PCA-motor (*p* < 0.001). In the putamen, evidence for a left-right difference in volume was absent in PCA-motor, weak in controls and strong in PCA-pure, with control and PCA-pure groups both showing greater volumes on the right. Evidence for a difference in the magnitude of putaminal asymmetry was absent for controls versus PCA-pure, weak for controls versus PCA-motor (*p* = 0.057) and strong for PCA-motor versus PCA-pure (*p* = 0.008).

### 3.6 Effect size analysis

Large effect sizes for the difference between PCA-motor and PCA-pure were found in the right thalamus and right putamen. Medium effect sizes were found in the right parietal, right temporal, and right central ROIs. Small effect sizes were found in right frontal, left parietal, left central and bilateral occipital regions, left putamen, and left thalamus (Supplementary Table 2). These effect sizes represent greater atrophy in the PCA-motor group than the PCA-pure group except for the left parietal and left occipital regions where atrophy was greater in the PCA-pure group.

## 4 Discussion

This study demonstrates an important overlap syndrome of PCA and CBS and reveals significant differences in the clinical and neuroimaging profiles of PCA patients with and without the core clinical features of CBS. All patients participating in the study met the originally proposed clinical criteria for PCA, of which 13 (30%) also showed prominent asymmetrical limb rigidity. Limb apraxia was evident on clinical examination in both the PCA-motor and PCA-pure groups but was seen more frequently, and was more often observed to be asymmetrical, in the PCA-motor group. This difference in asymmetry of apraxia between the groups was confirmed in the subgroup who underwent standardized assessment with the ABA-2,3A. Myoclonus was also seen in both groups but was more often asymmetrical in the PCA-motor group. Rest tremor and alien limb phenomena were only observed in the PCA-motor group. Other than a trend toward greater impairment on gesture production tests of praxis, no significant difference in neuropsychological measures was observed between the PCA-motor and PCA-pure groups. Clearly, the presence of prominent limb rigidity may impair an individual's ability to produce hand gestures so it is perhaps not surprising that more prominent and asymmetrical apraxia was observed in the PCA-motor group, where rigidity was used to define group membership. Teasing out the relative contribution of different motor signs can be difficult; myoclonus, tremor, and alien limb phenomena may also impact upon gesture production. The different motor signs should perhaps, therefore, be considered less as independent observations and more as a constellation of features that contribute to difficulty using the affected limb in a significant proportion of PCA patients in our cohort.

Neuroimaging analyses revealed overlapping but distinct patterns of tissue loss in the PCA-motor and PCA-pure groups. Both gray and white matter volume and cortical thickness techniques revealed more asymmetry in the PCA-motor than the PCA-pure group compared with controls, with more pronounced atrophy and reductions in thickness in the right hemisphere in the PCA-motor group. The regions found to show the greatest difference in VBM between these groups were the frontoparietal operculum, supramarginal gyrus, and middle part of the cingulate gyrus, all anterior to the maxima identified in the patient-control comparisons. These differences could not be attributed to age or disease duration and suggest a greater spatial extent of atrophy in the PCA-motor than PCA-pure group. The cortical thickness analysis showed a similar trend toward right-sided atrophy, and particularly emphasized the involvement of the perirolandic sensory and motor cortices (bilaterally but more on the right) in the PCA-motor group. Subcortical region of interest volumetric analysis revealed lower volumes of putamen and thalamus in the PCA-motor group compared with the PCA-pure group and healthy controls, an effect that was the strongest in the right hemisphere.

Asymmetry, with greater atrophy in the right hemisphere, was a prominent and distinctive feature of the PCA-motor group and may underlie the left upper limb motor features that were observed in these patients. The results are consistent with the hypothesis that CBS signs in PCA reflect atrophy of contralateral sensorimotor cortices. The data extend the hypothesis by demonstrating that such signs are also associated with greater tissue loss in subcortical structures, namely the putamen and thalamus. The thalamus and putamen have been found to undergo significant atrophy in AD (De Jong et al., 2008), however, their differential involvement in atypical AD phenotypes has not, to our knowledge, been systematically evaluated. Thalamic and basal ganglia volume loss on MRI has also been identified in CBS, however, does not appear to differentiate between CBS cases with underlying CBD or AD pathology (Josephs et al., 2010). In our study, the control and PCA-pure groups both showed greater volumes in the right thalamus and putamen. This normal asymmetry was lost in the PCA-motor group, implying that these structures had undergone a greater degree of atrophy in the right hemisphere. It seems likely that the prominent motor features in the PCA-motor group reflect dysfunction of a whole network of areas involved in the planning, coordination, and execution of movement. This includes the primary motor and somatosensory cortices, premotor and supplementary motor areas, and the posterior parietal and deep subcortical structures with which they connect.

It was striking that the asymmetric motor features affected the left side in all subjects in the PCA-motor group. Interestingly, the right side was involved more than the left in a series of CBS patients presenting with language, behavioral, or motor symptoms, with left hemispheric atrophy a frequent finding (Kertesz et al., 2000). Thus, it may be that our finding of motor features affecting the left side only reflects sampling bias in terms of the patients that are referred and labeled as having PCA. Patients with predominantly right hemisphere deficits affecting their vision may be more likely to present to a cognitive neurologist and be diagnosed with PCA than those with predominantly left hemisphere deficits such as progressive apraxia or dysgraphia. The latter type of patient is perhaps more likely to be referred to a movement disorders specialist and, if motor features are present (which would likely show a right limb predominance), be diagnosed with CBS. Supporting this hypothesis, a predominantly right-sided pattern of cortical atrophy has been reported in prior imaging studies of PCA (Lehmann et al., 2011a, Whitwell et al., 2007), and it has been proposed that this may be because of the relatively high proportion of patients with predominantly right hemisphere deficits in most PCA cohorts (Lehmann et al., 2011a). Our data showed no difference in the symmetry of cortical thickness between controls and the PCA-pure group in any cortical ROI. This perhaps suggests that asymmetry in PCA group studies may also be driven by the subset of subjects with additional features of CBS. Despite the difference in asymmetry between the PCA-motor and PCA-pure groups there was considerable overlap in their overall atrophy and cortical thickness patterns, with both groups showing the greatest differences from controls in occipital and parietal regions. It is therefore not surprising that apraxia and myoclonus were observed in both groups, as parietal lobe lesions may cause both of these clinical signs (Fahn et al., 2011).

There is significant pathological overlap in the etiology of PCA and CBS, with both potentially being caused by underlying AD, CBD, DLB, or CJD. Some clinicopathological studies investigating CBS patients with underlying AD or CBD have indicated that behavioral and/or frontal symptoms such as early personality change predict CBD pathology whereas impairment of visuospatial skills and memory, young age at onset and myoclonus are associated with AD. The presence of asymmetrical extrapyramidal signs, apraxia, or alien limb phenomena has not generally appeared to differentiate between CBS-CBD and CBS-AD (Hassan et al., 2011, Hu et al., 2009, Lee et al., 2011, Shelley et al., 2009). In PCA, which is characterized by prominent visuospatial impairment and in which young age at onset, myoclonus, and preservation of personality are commonly seen, additional features of CBS may still therefore associate with AD pathology. Contrary to the traditional view, it is in fact CBD that often appears to present without motor symptoms (with a frontal cognitive and/or behavioral syndrome) (Lee et al., 2011) or with motor features that are symmetrical (Hassan et al., 2010). In our cohort, AD pathology was confirmed in the 2 PCA-motor patients who underwent postmortem examination and was suggested by an AD-like CSF profile in the 2 other PCA-motor patients who underwent LP. The finding of additional LBP in the PCA-motor case is intriguing, particularly given this subject's history of extracampine hallucinations, and raises the possibility that concomitant LBP may influence the phenotype of AD. Additional LBP was found in 2 of the 5 pathologically confirmed CBS-AD patients reported by Josephs et al. (2010), one of whom developed visual hallucinations 6 years into her illness. Two of the 6 patients in the Tang-Wai PCA series with Parkinsonism had AD pathology with concomitant LBP; both subjects also had visual hallucinations, although neither the hallucinations nor Parkinsonism were present initially (Tang-Wai et al., 2004). Another 2 of the subjects in this series had CBD at postmortem, having shown signs of asymmetrical Parkinsonism and apraxia during life, demonstrating that PCA with motor features can be associated with non-AD pathology too. This has also been suggested by a previous CSF study, in which 1 of the 3 PCA subjects with asymmetric motor features had an AD-like CSF profile but CSF was normal for the other 2 (Seguin et al., 2011). Larger clinicopathological series will be needed to investigate whether AD with LBP does associate with motor features in PCA and to ascertain how frequently the phenotype is caused by CBD.

There is some evidence that imaging signatures may help to differentiate CBS subjects with pathologically confirmed AD (CBS-AD) from those with underlying CBD pathology. Greater atrophy of temporoparietal cortex and precuneus has been observed in patients with CBS-AD whereas CBS-CBD patients demonstrated more frontal atrophy (Josephs et al., 2010, Whitwell et al., 2010). CBS patients with an AD-like CSF profile have also shown greater hypoperfusion in precuneus and posterior cingulate cortex on single photon emission computed tomography scans than CBS patients with non-AD like CSF (Borroni et al., 2011). In our study, the fact that indirect (vs. controls) and direct comparisons between the PCA-motor and PCA-pure groups revealed predominant impairment of parietal and occipital but not more frontal regions supports the idea that the group differences reflect heterogeneity within the PCA syndrome, of which AD remains the commonest underlying cause, rather than suggesting that CBD pathology accounts for the PCA-motor phenotype.

The pathological overlap in the etiology of PCA and CBS emphasizes the need for improved clarity as to the clinical overlap between the 2 syndromes. Considering current clinical criteria for PCA (Mendez et al., 2002, Tang-Wai et al., 2004) and CBS (Boeve et al., 2003, Lang et al., 1994, Mathew et al., 2012), considerable overlap is evident meaning that some patients may fulfill criteria for both syndromes, as has been illustrated by a recent case report (Giorelli et al., 2014). This is particularly apparent in more recent CBS criteria, which have emphasized early cognitive change in executive function, language, memory, and/or visuospatial processing. For example, the modified Bak and Hodges (Cambridge) criteria for CBS could be met by PCA patients with insidious onset and no sustained levodopa response (mandatory criteria), limb apraxia and language impairment (major features) and a combination of at least 2 of alien limb phenomenon, cortical sensory loss, dyscalculia, and visual dysfunction (Mathew et al., 2012). Although typically considered a progressive visual syndrome, anomia, and phonological processing deficits are common early features in PCA (Benson et al., 1988, Crutch et al., 2013a, McMonagle et al., 2006). Conversely, patients fulfilling CBS criteria may also fulfill PCA criteria, which either have no exclusion criteria (Mendez et al., 2002) or exclude only early Parkinsonism (Tang-Wai et al., 2004). Thus, individuals diagnosed with CBS who have visual complaints and signs such as myoclonus, limb apraxia, alien limb phenomenon, and cortical sensory loss would still meet current criteria for PCA (Rajagopal et al., 2012).

Clarifying the relationship between PCA and CBS would have a number of implications for diagnosis, management, and research. First, although the presence of linguistic and executive dysfunction in CBS patients may not distinguish underlying pathologies (Lee et al., 2011), the presence of cortical visual dysfunction, and other posterior cortical deficits may weight the diagnostic probabilities toward AD. Consistent with this view, Rabinovici and Miller (2012) have speculated that some features within the modified Cambridge criteria for CBS such as visuospatial dysfunction and myoclonus may be predictive of AD. There is some support for this idea from a recent study of CBS patients with amyloid imaging using Pittsburgh compound B, which demonstrated greater visuospatial deficits in those with evidence of AD (amyloid) pathology than in those who were Pittsburgh compound B-negative (Burrell et al., 2013). Second, the current evidence of CBS-like motor features in a substantial proportion of PCA patients argues against any tendency to withhold currently available AD therapeutic treatments such as acetylcholinesterase inhibitors to PCA patients with these signs. Conversely, CBS patients presenting to movement disorders specialists should be examined closely for posterior cortical deficits and, if the phenotype suggests AD, offered appropriate treatment. The vast majority of patients with PCA have underlying AD (de Souza et al., 2011, Renner et al., 2004), and there is no evidence to date that the co-occurrence of CBS-like signs makes that diagnosis any less likely. In fact, the pathological and CSF data in this study, although limited, were consistent with an underlying diagnosis of AD in all the PCA-motor patients for whom this information was available. Third, although a syndromic classification could be adequate for some types of research study (e.g., brain-behavior correlations or behavioral interventions), other investigations will need direct consideration of probable underlying pathology (e.g., clinical trials of protein-specific therapies). Finally, the identification of CBS-like signs in patients who fulfill criteria for PCA has a bearing upon how inclusively or exclusively PCA should be defined in any future consensus criteria; for instance, should the term PCA only be applied to patients with predominant visual dysfunction (as specified by existing clinical criteria e.g., Mendez et al., 2002, Tang-Wai et al., 2004) or should the syndrome encompass patients with progressive deterioration in other cognitive domains such as calculation, spelling, and praxis (Aharon-Peretz et al., 1999, De Renzi, 1986, Green et al., 1995, Seguin et al., 2011, Snowden et al., 2007). Inclusivity is intuitively appealing given that the term PCA refers to the locus of focal atrophy rather than a particular set of clinical features. However, it might come at the cost of reduced specificity (in both syndromic and pathological terms) given reports of posterior cortical presentations with non-AD, non-CBD etiologies, for example CBS caused by a progranulin mutation presenting as progressive apraxic agraphia (Passov et al., 2011).

To our knowledge, this is the first study to compare directly the clinical and neuroimaging features of PCA patients with and without the core motor features of CBS. One limitation of the study is the relatively small number of subjects, which may explain the finding that although volumetric and cortical thickness asymmetries were evident at the level of control-patient comparisons and between-patient group percentage effect maps, direct comparisons between the 2 groups did not survive correction for multiple comparisons. However, this is a common problem when studying relatively rare degenerative conditions (Whitwell et al., 2007) and may be rectified with larger samples (Lehmann et al., 2011a). The MRI scans used in this study were acquired from scanners of different field strengths, which have the potential to introduce bias. However, the groups were well matched in terms of the proportion of scans acquired on each scanner, and scanner was included as a covariate in the imaging analyses to avoid an influence upon the results. Another limitation of the study was that detailed neuropsychological data and a standardized clinical assessment of limb apraxia were only available in a subset of patients. Although the neuropsychological results revealed a trend toward lower performance in gesture production in the PCA-motor group, further study should investigate the cognitive correlates of PCA with features of CBS in more detail. Despite only having been performed in a small subgroup of patients, the apraxia battery demonstrated significantly more asymmetry and a greater severity of left upper limb apraxia in the PCA-motor compared with PCA-pure group. This highlights the value of applying standardized clinical assessment tools to investigate neurological signs, such as the ABA-2 to evaluate apraxia, and the importance of assessing the left and right side separately in a condition like PCA. The retrospective nature of this study, with reliance on the recording of neurological signs by a number of different clinicians, were inevitable limitations which we attempted to overcome by only including patients with a clearly documented full neurological examination. Larger cohorts of patients, with clinicopathological correlation, will ultimately be required to further investigate the insights raised by this study. Multicenter collaboration may be needed to achieve this, particularly if the potential genetic factors underlying phenotypic heterogeneity in PCA are to be evaluated. The recent establishment of an international PCA Working Party represents a positive step in taking such collaboration forward (Crutch et al., 2013b), and in opening a dialog on the challenge of defining the syndrome of PCA, with appreciation of the motor as well as cognitive features that it may entail.

## Disclosure statement

The authors declare no actual or potential conflicts of interest.

## Figures and Tables

**Supplementary Table 1:** Division of 34 Desikan regions into 5 groups

Region of interest	Desikan region
Central	Paracentral
Central	Postcentral
Central	Precentral
Central	Posterior cingulate^a^
Frontal	Caudal anterior cingulate
Frontal	Caudal middle frontal
Frontal	Frontal pole
Frontal	Lateral orbitofrontal
Frontal	Medial orbitofrontal
Frontal	Pars opercularis
Frontal	Pars orbitalis
Frontal	Pars triangularis
Frontal	Rostral anterior cingulate
Frontal	Rostral middle frontal
Frontal	Superior frontal
Occipital	Lateral occipital
Occipital	Lingual
Occipital	Pericalcarine
Occipital	Cuneus
Parietal	Inferior parietal
Parietal	Isthmus cingulate
Parietal	Precuneus
Parietal	Superior parietal
Parietal	Supramarginal
Temporal	Bankssts
Temporal	Entorhinal
Temporal	Fusiform
Temporal	Inferior temporal
Temporal	Insula
Temporal	Middle temporal
Temporal	Parahippocampal
Temporal	Superior temporal
Temporal	Temporal pole
Temporal	Transverse temporal

a The region labeled posterior cingulate in the Desikan atlas (Desikan et al., 2006) is directly inferior to the paracentral lobule, rather than the posterior-most end of the cingulate, and was therefore placed in the central region of interest.

## References

[bib1] Aharon-Peretz J., Israel O., Goldsher D., Peretz A. (1999). Posterior cortical atrophy variants of Alzheimer’s disease. Demen. Geriatr. Cogn. Disord..

[bib2] Armstrong M.J., Litvan I., Lang A.E., Bhatia K.P., Borroni B., Boxer A.L., Dickson D.W., Grossman M., Hallett M., Josephs K.A., Kertesz A., Lee S.E., Miller B.L., Reich S.G., Riley D.E., Tolosa E., Tröster A.I., Vidailhet M., Weiner W.J. (2013). Criteria for the diagnosis of corticobasal degeneration. Neurology.

[bib3] Ashburner J. (2007). A fast diffeomorphic image registration algorithm. Neuroimage.

[bib4] Ashburner J., Friston K.J. (2005). Unified segmentation. Neuroimage.

[bib5] Ashburner J., Friston K.J. (2009). Computing average shaped tissue probability templates. Neuroimage.

[bib6] Baxter D.M., Warrington E.K. (1994). Measuring dysgraphia: a graded difficulty spelling test. Behav. Neurol..

[bib7] Benson D.F., Davis R.J., Snyder B.D. (1988). Posterior cortical atrophy. Arch. Neurol..

[bib8] Bian H., Van Swieten J.C., Leight S., Massimo L., Wood E., Forman M., Moore P., de Koning I., Clark C.M., Rosso S., Trojanowski J., Lee V.M., Grossman M. (2008). CSF biomarkers in frontotemporal lobar degeneration with known pathology. Neurology.

[bib9] Blennow K., Hampel H. (2003). CSF markers for incipient Alzheimer’s disease. Lancet Neurol..

[bib10] Boeve B.F., Lang A.E., Litvan I. (2003). Corticobasal degeneration and its relationship to progressive supranuclear palsy and frontotemporal dementia. Ann. Neurol..

[bib11] Boeve B.F., Maraganore D.M., Parisi J.E., Ahlskog J.E., Graff-Radford N., Caselli R.J., Dickson D.W., Kokmen E., Petersen R.C. (1999). Pathologic heterogeneity in clinically diagnosed corticobasal degeneration. Neurology.

[bib12] Borroni B., Premi E., Agosti C., Alberici A., Cerini C., Archetti S., Lanari A., Paghera B., Lucchini S., Caimi L., Padovani A. (2011). CSF Alzheimer’s disease-like pattern in corticobasal syndrome: evidence for a distinct disorder. J. Neurol. Neurosurg. Psychiatry.

[bib13] Braak H., Braak E. (1995). Staging of Alzheimer's disease-related neurofibrillary changes. Neurobiol. Aging.

[bib14] Burrell J.R., Hornberger M., Villemagne V.L., Rowe C.C., Hodges J.R. (2013). Clinical profile of PiB-positive corticobasal syndrome. PLoS One.

[bib15] Chung M.K., Worsley K.J., Nacewicz B.M., Dalton K.M., Davidson R.J. (2010). General multivariate linear modelling of surface shapes using SurfStat. Neuroimage.

[bib16] Crutch S.J., Rossor M.N., Warrington E.K. (2007). The quantitative assessment of apraxic deficits in Alzheimer’s disease. Cortex.

[bib17] Crutch S.J., Lehmann M., Gorgoraptis N., Kaski D., Ryan N., Husain M., Warrington E.K. (2011). Abnormal visual phenomena in posterior cortical atrophy. Neurocase.

[bib18] Crutch S.J., Lehmann M., Schott J.M., Rabinovici G.D., Rossor M.N., Fox N.C. (2012). Posterior cortical atrophy. Lancet Neurol..

[bib19] Crutch S.J., Lehmann M., Warren J.D., Rohrer J.D. (2013). The language profile of posterior cortical atrophy. J. Neurol. Neurosurg. Psychiatry.

[bib20] Crutch S., Schott J., Rabinovici G., Boeve B., Cappa S., Dubois B., Graff-Radford N.R., Krolak-Salmon P., Lehmann M., Mendez M.F., Pijnenburg Y., Ryan N.S., Scheltens P., Shakespeare T., Tang-Wai D.F., van der Flier W.M., Bain L., Carrillo M.C., Fox N.C. (2013). Shining a light on posterior cortical atrophy. Alzheimer’s Dement..

[bib21] Dabul B. (2000).

[bib22] Dale A.M., Fischl B., Sereno M.I. (1999). Cortical surface-based analysis. I. Segmentation and surface reconstruction. Neuroimage.

[bib23] De Jong L.W., Van der Hiele K., Veer I.M., Houwing J.J., Westendorp R.G.J., Bollen E.L., de Bruin P.W., Middelkoop H.A., van Buchem M.A., van der Grond J. (2008). Strongly reduced volumes of putamen and thalamus in Alzheimer’s disease: an MRI study. Brain.

[bib24] De Renzi E. (1986). Slowly progressive visual agnosia or apraxia without dementia. Cortex.

[bib25] Desikan R.S., Ségonne F., Fischl B., Quinn B.T., Dickerson B.C., Blacker D., Buckner R.L., Dale A.M., Maguire R.P., Hyman B.T., Albert M.S., Killiany R.J. (2006). An automated labeling system for subdividing the human cerebral cortex on MRI scans into gyral based regions of interest. Neuroimage.

[bib26] de Souza L.C., Corlier F., Habert M.O., Uspenskaya O., Maroy R., Lamari F., Chupin M., Lehéricy S., Colliot O., Hahn-Barma V., Samri D., Dubois B., Bottlaender M., Sarazin M. (2011). Similar amyloid-β burden in posterior cortical atrophy and Alzheimer's disease. Brain.

[bib27] Dickson D.W., Bergeron C., Chin S.S., Duyckaerts C., Horoupian D., Ikeda K., Jellinger K., Lantos P.L., Lippa C.F., Mirra S.S., Tabaton M., Vonsattel J.P., Wakabayashi K., Litvan I. (2002). Office of rare diseases neuropathologic criteria for corticobasal degeneration. J. Neuropathol. Exp. Neurol..

[bib28] Fahn S., Jankovic J., Hallett M. (2011).

[bib29] Fischl B., Dale A.M. (2000). Measuring the thickness of the human cerebral cortex from magnetic resonance images. Proc. Natl. Acad. Sci. U.S.A.

[bib30] Folstein M.F., Folstein S.E., McHugh P.R. (1975). “Mini-mental state”. A practical method for grading the cognitive state of patients for the clinician. J. Psychiatr. Res..

[bib81] Giorelli M., Losignore N.A., Bagnoli J., Difazio P., Zimatore G.B. (2014). The progression of posterior cortical atrophy to corticobasal syndrome: lumping or splitting neurodegenerative diseases?. Tremor Other Hyperkinet. Mov. (N.Y.).

[bib31] Graham N.L., Bak T.H., Hodges J.R. (2003). Corticobasal degeneration as a cognitive disorder. Mov. Disord..

[bib32] Green R.C., Goldstein F.C., Mirra S.S., Alazraki N.P., Baxt J.L., Bakay R.A. (1995). Slowly progressive apraxia in Alzheimer’s disease. J. Neurol. Neurosurg. Psychiatry.

[bib33] Grimes D.A., Lang A.E., Bergeron C.B. (1999). Dementia as the most common presentation of cortical-basal ganglionic degeneration. Neurology.

[bib34] Hammers A., Allom R., Koepp M.J., Free S.L., Myers R., Lemieux L., Mitchell T.N., Brooks D.J., Duncan J.S. (2003). Three-dimensional maximum probability atlas of the human brain, with particular reference to the temporal lobe. Hum. Brain Mapp..

[bib35] Hassan A., Whitwell J.L., Boeve B.F., Jack C.R., Parisi J.E., Dickson D.W., Josephs K.A. (2010). Symmetric corticobasal degeneration (S-CBD). Parkinsonism Relat. Disord..

[bib36] Hassan A., Whitwell J.L., Josephs K.A. (2011). The corticobasal syndrome-Alzheimer’s disease conundrum. Expert Rev. Neurother..

[bib37] Hu W.T., Rippon G.W., Boeve B.F., Knopman D.S., Petersen R.C., Parisi J.E., Josephs K.A. (2009). Alzheimer’s disease and corticobasal degeneration presenting as corticobasal syndrome. Mov. Disord..

[bib38] Jackson M., Warrington E.K. (1986). Arithmetic skills in patients with unilateral cerebral lesions. Cortex.

[bib39] Josephs K.A., Petersen R.C., Knopman D.S., Boeve B.F., Whitwell J.L., Duffy J.R., Parisi J.E., Dickson D.W. (2006). Clinicopathologic analysis of frontotemporal and corticobasal degenerations and PSP. Neurology.

[bib40] Josephs K.A., Whitwell J.L., Boeve B.F., Knopman D.S., Petersen R.C., Hu W.T., Parisi J.E., Dickson D.W., Jack C.R. (2010). Anatomical differences between CBS-corticobasal degeneration and CBS-Alzheimer’s disease. Mov. Disord..

[bib41] Kas A., de Souza L.C., Samri D., Bartolomeo P., Lacomblez L., Kalafat M., Migliaccio R., Thiebaut de Schotten M., Cohen L., Dubois B., Habert M.O., Sarazin M. (2011). Neural correlates of cognitive impairment in posterior cortical atrophy. Brain.

[bib42] Kennedy J., Lehmann M., Sokolska M.J., Archer H., Warrington E.K., Fox N.C., Crutch S.J. (2012). Visualizing the emergence of posterior cortical atrophy. Neurocase.

[bib43] Kertesz A., Martinez-Lage P., Davidson W., Munoz D.G. (2000). The corticobasal degeneration syndrome overlaps progressive aphasia and frontotemporal dementia. Neurology.

[bib44] Lang A., Riley D., Bergeron C. (1994).

[bib45] Lee S.E., Rabinovici G.D., Mayo M.C., Wilson S.M., Seeley W.W., DeArmond S.J., Huang E.J., Trojanowski J.Q., Growdon M.E., Jang J.Y., Sidhu M., See T.M., Karydas A.M., Gorno-Tempini M.L., Boxer A.L., Weiner M.W., Geschwind M.D., Rankin K.P., Miller B.L. (2011). Clinicopathological correlations in corticobasal degeneration. Ann. Neurol..

[bib46] Lehmann M., Crutch S.J., Ridgway G.R., Ridha B.H., Barnes J., Warrington E.K., Rossor M.N., Fox N.C. (2011). Cortical thickness and voxel-based morphometry in posterior cortical atrophy and typical Alzheimer’s disease. Neurobiol. Aging.

[bib47] Lehmann M., Barnes J., Ridgway G.R., Wattam-Bell J., Warrington E.K., Fox N.C., Crutch S.J. (2011). Basic visual function and cortical thickness patterns in posterior cortical atrophy. Cereb. Cortex.

[bib48] Lehmann M., Barnes J., Ridgway G.R., Ryan N.S., Warrington E.K., Crutch S.J., Fox N.C. (2012). Global grey matter changes in posterior cortical atrophy: a serial imaging study. Alzheimers Dement..

[bib49] Leung K.K., Barnes J., Ridgway G.R., Bartlett J.W., Clarkson M.J., Macdonald K., Schuff N., Fox N.C., Ourselin S., Alzheimer's Disease Neuroimaging Initiative (2010). Automated cross-sectional and longitudinal hippocampal volume measurement in mild cognitive impairment and Alzheimer’s disease. Neuroimage.

[bib50] Leung K.K., Barnes J., Modat M., Ridgway G.R., Bartlett J.W., Fox N.C., Ourselin S., Alzheimer's Disease Neuroimaging Initiative (2011). Brain MAPS: an automated, accurate and robust brain extraction technique using a template library. Neuroimage.

[bib51] Ling H., O’Sullivan S.S., Holton J.L., Revesz T., Massey L.A., Williams D.R., Paviour D.C., Lees A.J. (2010). Does corticobasal degeneration exist? A clinicopathological re-evaluation. Brain.

[bib52] Mathew R., Bak T.H., Hodges J.R. (2012). Diagnostic criteria for corticobasal syndrome: a comparative study. J. Neurol. Neurosurg. Psychiatry.

[bib53] McMonagle P., Deering F., Berliner Y., Kertesz A. (2006). The cognitive profile of posterior cortical atrophy. Neurology.

[bib54] Mendez M.F., Ghajarania M., Perryman K.M. (2002). Posterior cortical atrophy: clinical characteristics and differences compared to Alzheimer's disease. Demen. Geriatr. Cogn. Disord..

[bib55] Murray R., Neumann M., Forman M.S., Farmer J., Massimo L., Rice A., Miller B.L., Johnson J.K., Clark C.M., Hurtig H.I., Gorno-Tempini M.L., Lee V.M., Trojanowski J.Q., Grossman M. (2007). Cognitive and motor assessment in autopsy-proven corticobasal degeneration. Neurology.

[bib56] Passov V., Gavrilova R.H., Strand E., Cerhan J.H., Josephs K.A. (2011). Sporadic corticobasal syndrome with progranulin mutation presenting as progressive apraxic agraphia. Arch. Neurol..

[bib57] Rabinovici G.D., Miller B.L. (2012). Corticobasal syndrome: overcoming the artificial divide between disorders of cognition and movement. J. Neurol. Neurosurg. Psychiatry.

[bib58] Rajagopal R., Bateman R., Van Stavern G.P. (2012). Visual involvement in corticobasal syndrome. J. Neuroophthalmol..

[bib59] Rebeiz J.J., Kolodny E.H., Richardson E.P. (1968). Corticodentatonigral degeneration with neuronal achromasia. Arch. Neurol..

[bib60] Renner J.A., Burns J.M., Hou C.E., McKeel D.W., Storandt M., Morris J.C. (2004). Progressive posterior cortical dysfunction: a clinicopathologic series. Neurology.

[bib61] Ridgway G.R., Omar R., Ourselin S., Hill D.L., Warren J.D., Fox N.C. (2009). Issues with threshold masking in voxel-based morphometry of atrophied brains. Neuroimage.

[bib62] Ridgway G.R., Lehmann M., Barnes J., Rohrer J.D., Warren J.D., Crutch S.J., Fox N.C. (2012). Early-onset Alzheimer's disease clinical variants: multivariate analyses of cortical thickness. Neurology.

[bib63] Schneider J.A., Watts R.L., Gearing M., Brewer R.P., Mirra S.S. (1997). Corticobasal degeneration: neuropathologic and clinical heterogeneity. Neurology.

[bib64] Seguin J., Formaglio M., Perret-Liaudet A., Quadrio I., Tholance Y., Rouaud O., Thomas-Anterion C., Croisile B., Mollion H., Moreaud O., Salzmann M., Dorey A., Bataillard M., Coste M.H., Vighetto A., Krolak-Salmon P. (2011). CSF biomarkers in posterior cortical atrophy. Neurology.

[bib65] Shakespeare T.J., Yong K.X., Frost C., Kim L.G., Warrington E.K., Crutch S.J. (2013). Scene perception in posterior cortical atrophy: categorization, description and fixation patterns. Front. Hum. Neurosci..

[bib66] Shelley B.P., Hodges J.R., Kipps C.M., Xuereb J.H., Bak T.H. (2009). Is the pathology of corticobasal syndrome predictable in life?. Mov.t Disord..

[bib67] Snowden J.S., Stopford C.L., Julien C.L., Thompson J.C., Davidson Y., Gibbons L., Pritchard A., Lendon C.L., Richardson A.M., Varma A., Neary D., Mann D. (2007). Cognitive phenotypes in Alzheimer’s disease and genetic risk. Cortex.

[bib68] Tang-Wai D.F., Josephs K.A., Boeve B.F., Dickson D.W., Parisi J.E., Petersen R.C. (2003). Pathologically confirmed corticobasal degeneration presenting with visuospatial dysfunction. Neurology.

[bib69] Tang-Wai D.F., Josephs K.A., Boeve B.F., Petersen R.C., Parisi J.E., Dickson D.W. (2003). Coexistent Lewy body disease in a case of “visual variant of Alzheimer’s disease”. J. Neurol. Neurosurg. Psychiatry.

[bib70] Tang-Wai D.F., Graff-Radford N.R., Boeve B.F., Dickson D.W., Parisi J.E., Crook R., Caselli R.J., Knopman D.S., Petersen R.C. (2004). Clinical, genetic, and neuropathologic characteristics of posterior cortical atrophy. Neurology.

[bib71] Warfield S.K., Zou K.H., Wells W.M. (2004). Simultaneous truth and performance level estimation (STAPLE): an algorithm for the validation of image segmentation. IEEE Trans. Med. Imaging.

[bib72] Warrington E. (1996).

[bib73] Warrington E.K., James M. (1988). Visual apperceptive agnosia: a clinico-anatomical study of three cases. Cortex.

[bib74] Warrington E.K., James M. (1991).

[bib75] Warrington E., McKenna P., Orpwood L. (1998). Single word comprehension: a concrete and abstract word synonym test. Neuropsychol. Rehabil..

[bib76] Weiskopf N., Lutti A., Helms G., Novak M., Ashburner J., Hutton C. (2011). Unified segmentation based correction of R1 brain maps for RF transit field inhomogeneities (UNICORT). Neuroimage.

[bib77] Whitwell J.L., Jack C.R., Kantarci K., Weigand S.D., Boeve B.F., Knopman D.S., Drubach D.A., Tang-Wai D.F., Petersen R.C., Josephs K.A. (2007). Imaging correlates of posterior cortical atrophy. Neurobiol. Aging.

[bib78] Whitwell J.L., Jack C.R., Boeve B.F., Parisi J.E., Ahlskog J.E., Drubach D.A., Senjem M.L., Knopman D.S., Petersen R.C., Dickson D.W., Josephs K.A. (2010). Imaging correlates of pathology in corticobasal syndrome. Neurology.

[bib79] Witoonpanich P., Cash D.M., Shakespeare T.J., Yong K.X., Nicholas J.M., Omar R., Crutch S.J., Rossor M.N., Warren J.D. (2013). Olfactory impairment in posterior cortical atrophy. J. Neurol. Neurosurg. Psychiatry.

[bib80] Yong K.X., Warren J.D., Warrington E.K., Crutch S.J. (2013). Intact reading in patients with profound early visual dysfunction. Cortex.

