# Supplementary material for: Motor features in posterior cortical atrophy and their imaging correlates
Source: Neurobiol Aging. 2014 Dec;35(12):2845–57. doi: 10.1016/j.neurobiolaging.2014.05.028 (PMC4236588; doi:10.1016/j.neurobiolaging.2014.05.028)
Supplement: Supplementary Material [file mmc1.docx]

**Supplementary Material**

**MRI scan acquisition protocols**

Scans were acquired using either a 1.5T or 3T scanner. Three different 1.5T scanners were used.

***3T scanner – National Hospital for Neurology and Neurosurgery***

Scans were acquired using a Siemens Trio TIM 3T scanner using an MPRAGE sequence with a 256x256 acquisition matrix and 28.2-cm field of view to provide 208 contiguous 1.1mm slices in the sagittal plane; acquisition parameters: TE=2.9ms, TR=2200ms, TI=900ms. 18 healthy controls, 18 PCA-Pure and 7 PCA-Motor scans were acquired on this scanner.

***1.5T scanner – NMR group, Queen Square***

This scanner used an inversion recovery (IR)-prepared fast SPGR sequence with a 256x256 image matrix and a 24cm field of view. The scanner underwent a software upgrade in April 2004, which consisted of new gradient drivers, a new computer operating system (Linux platform) and a scanner operating software upgrade from v5.8 to v11.0. Scans acquired prior to the upgrade have the following parameters: readout interval repetition time TR=15ms; TE=5.4ms; TI=650ms; flip angle=15°. Scans obtained after the upgrade have the following parameters: readout interval TR=12ms; TE=5.2ms; TI=650ms; flip angle=13°. 5 healthy controls, 6 PCA-Pure and 4 PCA-Motor scans were acquired on this scanner. All controls, 2 PCA-Pure and 1 PCA-Motor patient were scanned prior to the software upgrade.

***1.5T scanner – Queen Square Imaging Centre***

Scans were acquired using a 256x224 image matrix with the field of view being 24cm. The scanning protocol used the following acquisition parameters: TE=2.3ms; TR=20ms; TI=450ms; flip angle=20°. 5 healthy controls, 4 PCA-Pure and 1 PCA-Motor scan was acquired on this scanner.

***1.5T scanner – National Hospital for Neurology and Neurosurgery***

This scanner used an IR-prepared SPGR sequence with a 256x256 image matrix and a field of view of 24cm; acquisition parameters: TE=6.3ms; readout interval TR=14.2ms; TI=650ms; flip angle=15°. 2 healthy controls, 3 PCA-Pure and 1 PCA-Motor scans were acquired on this scanner.

Supplementary Table 1. P values for linear regression effects and post hoc pairwise comparisons in cortical thickness and subcortical volume ROI analysis. Table shows p value (mean difference) for each of the pairwise comparisons performed. Shading indicates p<0.05.

|  | PCA-Pure vs  Control^1^ | PCA-Motor vs  Control^1^ | PCA-Motor vs  PCA-Pure^2^ | L vs R in controls^3^ | L vs R in PCA-Motor^3^ | L vs R in PCA-Pure^3^ | L vs R in controls compared to PCA-Motor^4^ | L vs R in controls compared to PCA-Pure^4^ | L vs R in PCA-Motor compared to PCA-Pure^5^ |
| --- | --- | --- | --- | --- | --- | --- | --- | --- | --- |
| **Cortical ROIs** |  |  |  |  |  |  |  |  |  |
| **All** | <0.001  (-0.25) | <0.001  (-0.33) | 0.730  (-0.03) | 0.388 (0.00) | <0.001  (-0.09) | 0.963 (0.00) | <0.001  (0.10) | 0.849  (0.00) | <0.001 (0.10) |
| **Parietal** | <0.001  (-0.40) | <0.001  (-0.41) | 0.978  (-0.01) | 0.043 (0.02) | <0.001  (-0.13) | 0.864 (0.00) | <0.001  (0.15) | 0.570  (0.02) | <0.001 (0.14) |
| **Frontal** | 0.013  (-0.08) | 0.030  (-0.11) | 0.826  (-0.02) | <0.001  (-0.06) | 0.007  (-0.08) | 0.006  (-0.04) | 0.630  (0.02) | 0.413  (-0.02) | 0.297 (0.03) |
| **Temporal** | <0.001  (-0.29) | <0.001  (-0.34) | 0.611  (-0.05) | 0.008 (0.03) | 0.006  (-0.08) | 0.228 (0.04) | <0.001  (0.11) | 0.863  (-0.01) | 0.008 (0.12) |
| **Occipital** | <0.001  (-0.27) | <0.001  (-0.26) | 0.455 (0.01) | <0.001 (0.04) | 0.004  (-0.09) | 0.155 (0.03) | <0.001  (0.13) | 0.520  (0.01) | 0.002 (0.11) |
| **Central** | <0.001  (-0.18) | <0.001  (-0.26) | 0.240  (-0.08) | 0.309  (-0.01) | <0.001  (-0.10) | 0.242  (-0.03) | <0.001  (0.09) | 0.550  (0.01) | 0.013 (0.07) |
| **Subcortical ROIs** |  |  |  |  |  |  |  |  |  |
| **All** | <0.001  (-509) | <0.001  (-848) | 0.009  (-339) | <0.001 (242) | 0.426  (-63) | <0.001 (221) | <0.001  (305) | 0.761  (21) | 0.006 (284) |
| **Caudate** | 0.002  (-411) | 0.002  (-417) | 0.998  (-6) | 0.614 (62) | 0.86  (-39) | 0.666  (-31) | 0.689  (101) | 0.514  (93) | 0.973  (8) |
| **Putamen** | 0.002  (-393) | 0.002  (-841) | 0.004  (-448) | 0.084 (129) | 0.304  (-87) | 0.003 (214) | 0.057  (216) | 0.406  (-85) | 0.008 (301) |
| **Thalamus** | <0.001  (-723) | <0.001  (-1,286) | 0.005  (-563) | <0.001 (535) | 0.464  (-63) | <0.001 (480) | <0.001  (598) | 0.697  (56) | <0.001 (543) |

1. Negative means represent reduced thickness in the PCA-Pure and PCA-Motor group than control group,

2. Negative means represent reduced thickness in the PCA-Motor group compared to the PCA-Pure group.

3. Negative means represent reduced thickness in the right hemisphere.

4. Positive means represent greater asymmetry (right thinner than left) in the PCA-Motor compared to control and PCA-Pure groups.

5. Positive means represent greater asymmetry (right thinner than left) in the PCA-Motor compared to PCA-Pure group.

Supplementary Table 2. Cohen's d measure of effect size for PCA-Motor vs. PCA-Pure (the two groups differ by d of a standard deviation, e.g. a d of 0.5 means they differ by half a SD, and is considered a medium effect size). Dark shading indicates large effect size, light shading indicates small effect size. The warm colours are used when thickness or volume is lower in PCA-Motor than PCA-Pure, cold colours show the opposite.

| **all cortical areas** | | **parietal** | | **frontal** | | | **temporal** | | **occipital** | | **central** | |
| --- | --- | --- | --- | --- | --- | --- | --- | --- | --- | --- | --- | --- |
| Left | Right | Left | Right | Left | Right | | Left | Right | Left | Right | Left | Right |
| 0.06 | 0.28 | 0.40 | 0.51 | 0.04 | 0.26 | | 0.03 | 0.58 | 0.45 | 0.36 | 0.23 | 0.70 |
|  |  |  |  |  |  | |  |  |  |  |  |  |
|  |  |  |  |  |  | |  |  |  | PCA-Motor less than PCA-Pure | | |
| **all subcortical areas** | | **caudate** | | **putamen** | | | **thalamus** | |  | large | medium | small |
| Left | Right | Left | Right | Left | | Right | Left | Right |  | PCA-Pure less than PCA-Motor | | |
| 0.20 | 0.41 | 0.00 | 0.01 | 0.47 | | 0.88 | 0.39 | 1.00 |  | large | medium | small |
